# Efficacy of ChatGPT in personalized glucose-lowering strategy development: a clinician-based comparative study

**DOI:** 10.3389/fendo.2026.1693381

**Published:** 2026-03-11

**Authors:** Yu Wang, Cheng-Lin Zhang, Huijuan Zhao, Chang Wang, Lin Guo, Pengfei Wei, Mingyue Jin, Aiping Li, Qiang Li, Hongyan Pan

**Affiliations:** 1 Department of Endocrinology and Metabolism, Shenzhen University General Hospital, Shenzhen, Guangdong, China; 2 Department of Biomedical Sciences, City University of Hong Kong, Hong Kong, Hong Kong SAR, China; 3 Department of Pathophysiology, Shenzhen University Medical School, Shenzhen, Guangdong, China; 4 Department of Nephrology, Institute of Nephrology, 2nd Affiliated Hospital of Hainan Medical University, Haikou, Hainan, China

**Keywords:** attending physicians, ChatGPT, comparative study, general practitioners, large language model, personalized glucose-lowering strategy

## Abstract

**Background:**

The increasing incidence of diabetes poses a significant burden on healthcare systems. Limited research exists on tools to assist providers in developing personalized glucose-lowering strategies, which could alleviate this pressure and enhance patient outcomes.

**Objective:**

This study aims to evaluate the capability of ChatGPT-4o in developing personalized glucose-lowering strategies for individuals with diabetes.

**Methods:**

First, an evaluation of ChatGPT-4o’s performance on China’s qualification examination for attending physicians in endocrinology. Second, a cross-sectional study was conducted, involving the comparison of glucose-lowering strategies formulated by ChatGPT-4o, general practitioners (GPs), and attending physicians (APs) in endocrinology for a set of 30 real-world diabetes cases. Three clinical experts scored blindly the reasonableness of each strategy on a scale, with stratification of cases into three complexity levels (A, B, and C) and evaluation of mean scores for each level.

**Results:**

ChatGPT-4o successfully passed all sections of the qualification examination with scores above the 60% threshold. In developing glucose-lowering strategies, ChatGPT-4o achieved a mean score comparable to GPs (82.24 ± 9.933 *vs* 79.83 ± 3.768; p = .317) but lower than APs (82.24 ± 9.933 *vs* 86.35 ± 4.142; p = .0467). Performance declined with increasing case complexity, with mean scores dropping from 89.90 ± 2.936 for simple cases (A-level) to 76.12 ± 11.93 for complex cases (C-level) (p <.0020).

**Conclusions:**

ChatGPT-4o performs reliably in generating glucose-lowering strategies for simpler diabetes cases, highlighting its potential to assist community health workers. However, its accuracy in complex cases, especially concerning medication contraindications, requires improvement.

## 1 Introduction

The rising prevalence of diabetes presents a substantial threat to global public health. It is projected that by 2045, diabetes will affect 783 million individuals worldwide (1), imposing significant economic and workload challenges on healthcare systems, general practitioners (GPs), and endocrinologist. Moreover, according to the 2021 International Diabetes Federation’s Diabetes Atlas, it is estimated that 44.7% of adults with diabetes remain undiagnosed, with over 81% of these individuals residing in low- and middle-income countries that have limited medical resources (1). The management of diabetes encompasses diabetes education, dietary interventions, exercise regimens, pharmacotherapy, and self-monitoring of blood glucose. However, chronic disease management demands significant time and energy from primary healthcare workers. Furthermore, the uneven distribution of medical resources often results in suboptimal management of many diabetic patients, particularly those in remote or low-income areas. These patients often lack access to specialized care from endocrinologists. The development of a personalized glucose-lowering strategy represents a pivotal aspect of effective diabetes management. Achieving this necessitates careful consideration of patient-specific factors, requiring healthcare workers to possess both a strong foundation in fundamental knowledge and extensive clinical expertise.

Artificial intelligence (AI) is a rapidly evolving field of science, particularly in light of recent developments in Large Language Models (LLMs). LLMs are developed using advanced natural language processing techniques, enabling them to recognize and comprehend the structure and semantics of human language. These models are capable of classifying texts based on content or intent and generating responses that are both contextually appropriate and coherent (2). Chat Generative Pre-Trained Transformer (ChatGTP) represents a notable milestone of AI development (3). ChatGPT-4o was developed by OpenAI and released in May 2024. The language model was trained on a corpus of textual data comprising web pages, articles, and books, up until October 2023.

As a representative of advanced AI, ChatGPT has demonstrated considerable potential both preclinically and clinically. Researchers assessed ChatGPT’s clinical reasoning abilities by testing it with questions from the U.S. Medical Licensing Examination (USMLE). Impressively, without any prior reinforcement or specific training, the technology managed to pass these exams with an accuracy rate of 60% (4, 5). While passing the examination is only a prerequisite of becoming a doctor, the potential applications of ChatGPT in medicine are currently being extensively researched. In fact, AI has shown great potential in the management of diabetes (6), including health education (7–9), AI-based diet recommendations (10, 11), intelligent personalized exercise prescription (12, 13), prediction and detection of hypoglycemia (14, 15), dosing strategies for insulin therapy (16) and screening for diabetes complications (17). However, there is scarce data on its reliability and accuracy in personalizing glucose-lowering strategies for clinical diabetic cases. In this study, we aimed to explore whether ChatGPT can accurately formulate glucose-lowering strategies for different types of clinical diabetic cases, with the aim of determining whether AI can effectively serve as an assistant to healthcare workers, support clinical decision-making, and identify its potential limitations for future enhancement.

## 2 Methods

**Figure 1:**
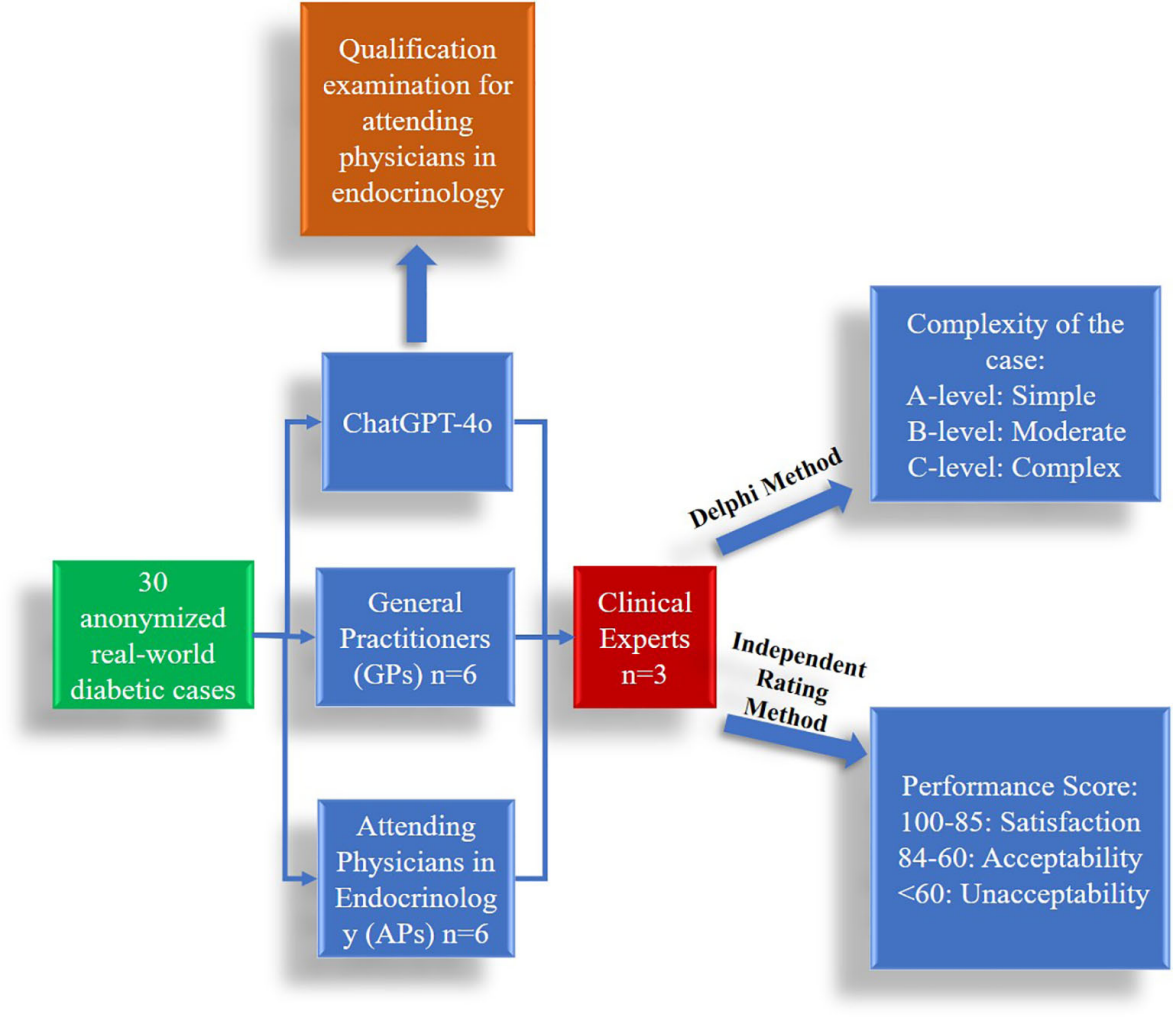
Study flow diagram.

**Figure 2:**
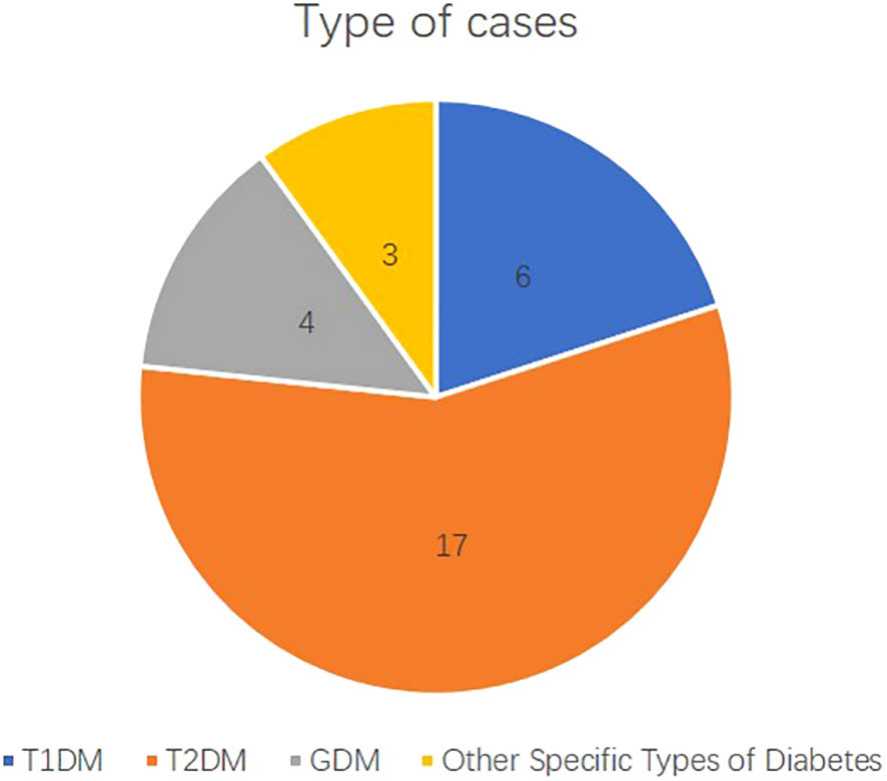
Type of cases. Distribution of different types of diabetes mellitus cases. The bar graph represents the number of cases for Type 1 Diabetes Mellitus (T1DM, n=6), Type 2 Diabetes Mellitus (T2DM, n=17), Gestational Diabetes Mellitus (GDM, n=4), and Other Specific Types of Diabetes (n=3).

This cross-sectional study was conducted in a large tertiary hospital in Guangdong Province, China, and included 30 anonymized real-world diabetic cases. Participants comprised six attending physicians (APs) in endocrinology from three major tertiary hospitals, six general practitioners (GPs) from five community health service centers, and three clinical experts in endocrinology from two major tertiary hospitals. This study follows Strengthening the Reporting of Observational Studies in Epidemiology (STROBE) reporting guideline. **Figure 1** illustrates the experimental procedure. **Figure 2** illustrates distribution of different types of diabetes mellitus cases. This study was reviewed by the Institutional Review Board of Shenzhen University General Hospital (SUGH), which determined that the use of de-identified clinical text data from the SUGH Information Commons does not constitute human subjects research and is therefore exempt from further ethical review and the requirement for informed consent. All data were processed through de-identification procedures in accordance with the Personal Information Protection Law of the People's Republic of China to ensure that no individual could be identified without additional information.

### 2.1 Qualification examination for attending physicians in endocrinology

In the beginning, we tested ChatGPT-4o on questions from the qualification examination for APs in endocrinology in China (qualification exam questions used in our study were all based on content from before 2023, which is well within the training data window of ChatGPT-4o), assessing its mastery of professional knowledge in internal medicine and endocrinology. The examination is divided into four sections: Fundamental Knowledge (100 single-choice questions), Relevant Professional Knowledge (100 single-choice questions), Professional Knowledge (100 single-choice questions), and Professional Practice Ability (70 multiple-choice questions, with multiple correct answers). The Fundamental Knowledge and Relevant Professional Knowledge sections focus on fundamental concepts and case analysis in internal medicine. The Professional Knowledge and Professional Practice Ability sections assess essential endocrinology knowledge and clinical competence. An accuracy rate of over 60% in each section of the exam is considered passing.

### 2.2 Evaluation of the ability of ChatGPT-4o, GPs, and APs to formulate personalized glucose-lowering strategies

After that, we assessed ChatGPT-4o’s clinical practice abilities and compared them with those of GPs working in community health centers and APs working in endocrine specialties in large general hospitals. For this analysis, we selected 30 real-world clinical diabetic cases admitted to the Department of Endocrinology and Metabolic Diseases at SUGH, a tertiary hospital, between 2021 and 2024. These cases included various types of diabetes, such as type 1 diabetes, type 2 diabetes, gestational diabetes and specific types of diabetes, covering both pediatric and adult populations. Brief cases information is presented in **Table 1**. To begin with, six GPs from five community health centers, six APs in endocrinology from three large tertiary general hospitals, and ChatGPT-4o were tasked with developing personalized glucose-lowering strategies tailored to the specific characteristics of these cases. The cases were categorized using a three-point scale, with categories A (n = 10), B (n = 9), and C (n = 11) representing progressively increasing levels of complexity, which were determined by three clinical experts in endocrinology from two hospitals, each with over 20 years of clinical experience and senior professional titles using the Delphi method. Complexity factors included advanced age, impaired liver or kidney function, intricate medical histories, and potential drug contraindications. The quality of the proposed glucose-lowering strategies was independently evaluated by three clinical experts, in accordance with the “China Guidelines for the Prevention and Treatment of Type 2 Diabetes (2023)” and the “ADA Standards of Care in Diabetes-2023”. The evaluation criteria included the safety and rationale of drug selection and combinations, dosage choices, management of side effects, and the presence of any drug contraindications (**Table 2**). After that, a designated individual standardized the responses from the three groups into comparable sentences to ensure consistency and to eliminate any potential influence of linguistic variations on the expert assessments. In the end, three clinical experts in endocrinology conducted a comprehensive evaluation and analysis of the quality and limitations of the responses provided by the GPs, APs, and ChatGPT-4o. The evaluation scores ranged from 0 to 100, with scores of 85 or above indicating satisfaction, meaning that the drug selection and combinations were appropriate, dosages were reasonable, and they met the patient’s personalized needs. Scores of 60 to 84 indicated acceptability, suggesting that while the drug selection was adequate, there may have been certain deficiencies, such as dosage or type, and it lacked personalization for the patient’s specific characteristics. Scores below 60 indicated unacceptability, highlighting significant deficiencies, such as inappropriate or unsafe medication choices. All participating clinicians and experts were blinded to the purpose of the study and the experimental group assignments. The specific inclusion criteria were as follows:

**Table 1:** Brief clinical characteristics of the study cohort with different diabetes subtypes.

ID	Age (years)	Sex	Disease duration	BMI (kg/m²)	HbA1c (%)	Type	Clinical characteristics
A19	35	F	1 week	23.02	6.5	GDM	Gestational diabetes mellitus
A21	28	F	1 day	28	7.5	GDM	Insulin resistance
A24	32	F	1 day	25	5.8	GDM	Mild GDM
B23	30	F	2 weeks	23	6.2	GDM	Hepatic impairment
A28	45	M	1 year	19.60	9.5	Other Specific Types of Diabetes	Pancreatogenic diabetes
C29	28	F	6 months	19.30	7.8	Other Specific Types of Diabetes	MODY3 (HNF1α mutation)
C30	38	F	1 year	18.4	8.9	Other Specific Types of Diabetes	Mitochondrial diabetes (m.3243A>G mutation)
A14	28	F	8 months	17.57	7	T1DM	New-onset diabetes
A17	6.75	M	20 days	15.1	12.7	T1DM	Pediatric case
B25	32	M	3 months	20.2	8.7	T1DM	LADA
B27	52	M	12 years	19.03	9.2	T1DM	Renal dysfunction
C22	25	F	3 days	19.5	7.8	T1DM	Fulminant type 1 diabetes during pregnancy
C26	14	F	1 month	16.8	12.5	T1DM	DKA
A11	33	M	1 year	37.62	8.8	T2DM	Mild hepatic dysfunction
A15	63	M	20 years	23.72	7.6	T2DM	Long-standing diabetes with poor islet function
A16	13	F	3 days	25.68	7.7	T2DM	Pediatric case
A18	26	M	1 month	22.79	12.5	T2DM	Diabetic ketosis
B12	33	M	3 days	22.03	6.6	T2DM	New-onset diabetes
B13	34	F	1 year	31.77	8	T2DM	New-onset diabetes
B3	65	F	2 days	27.18	6	T2DM	Family history of medullary thyroid carcinoma
B4	48	M	3 years	28.68	8	T2DM	Bladder cancer
B6	76	M	20 days	26.98	6.6	T2DM	Heart failure
B9	71	M	3 years	28.4	8	T2DM	Renal insufficiency
C1	34	M	1 year	28.87	7.8	T2DM	History of pancreatitis
C10	72	M	1 day	21.64	8.5	T2DM	Renal insufficiency
C2	43	M	2 years	31.7	6.7	T2DM	Hyperlipidemia
C20	81	F	3 years	19.8	8	T2DM	Pancreatic cancer with hepatic dysfunction
C5	43	M	3 years	25.49	7.9	T2DM	Gastrointestinal dysfunction
C7	34	M	1 month	57.7	6.3	T2DM	Severe obstructive sleep apnea with hypoxemia
C8	61	F	10 years	17.41	9.8	T2DM	Diabetic nephropathy

M, male; F, female.

**Table 2:** Evaluation criteria and weight distribution.

Evaluation criteria	Weight (%)	Description
Safety of drug selection and combinations	30%	Assessment of whether chosen drugs and their combinations pose minimal risks.
Rationale for drug selection	20%	Justification based on guidelines, evidence, or patient-specific factors.
Appropriateness of dosage choices	20%	Evaluation of dosing accuracy (e.g., age, weight, renal/hepatic function).
Management of side effects	20%	Documentation of monitoring and mitigation strategies for adverse effects.
Presence of drug contraindications	10%	Verification that no absolute contraindications were overlooked.

- GPs: those general practitioners working at community health service centers with 2–4 years of clinical experience and junior professional titles were included.
- APs: those from the endocrinology department of large tertiary general hospitals with 2–4 years of clinical practice and junior professional titles were included.
- Clinical experts: those from the endocrinology department of large tertiary general hospitals with more than 20 years of clinical practice and senior professional titles were included.

### 2.3 Statistical analysis

The statistical analysis for multiple-group comparisons among three groups was conducted using Brown-Forsythe and Welch ANOVA to account for potential heterogeneity of variances. *Post hoc* pairwise comparisons were performed using the Dunnett T3 test with adjustments for multiple testing to maintain the family-wise error rate at an acceptable level. The mean scores for each group, along with their standard deviations (SD), were reported to illustrate both the central tendency and the dispersion of the data. The significance level was set at p <.05 for all tests conducted, which were two-tailed. All statistical analyses were executed using Prism 8.0.1 software, ensuring the precision and reliability of the outcomes.

## 3 Results

### 3.1 Qualification examination for attending physicians in endocrinology

In our evaluation of ChatGPT-4o, we systematically assessed its performance using a series of test questions, with specific results detailed in **Supplementary Table 1**. As shown in **Table 3**, ChatGPT-4o, without any prior reinforcement or training, achieved scores in all four sections that exceeded the passing threshold, successfully meeting the requirements to pass the qualification examination for attending physicians in endocrinology.

**Table 3:** Scores of ChatGTP-4o for the qualification examination for attending physicians in endocrinology in China.

Proportion and item	Score	Accuracy rate (%)
Section 1 (Fundamental Knowledge)	84/100	84%
Section 2 (Relevant Professional Knowledge)	68/100	68%
Section 3 (Professional Knowledge)	84/100	84%
Section 4 (Professional Practice Ability)	49.7/70	71%

### 3.2 Evaluation of the ability of ChatGPT-4o, GPs, and APs to formulate personalized glucose-lowering strategies

The answer produced by ChatGPT-4o can be broadly classified into four main sections: lifestyle modifications, pharmacologic therapy, monitoring and follow-up, and additional considerations. To guarantee the precision of the study outcomes, we only assessed the pharmacologic therapy part. To ensure consistency across responses from three groups and minimize the impact of linguistic differences on expert evaluations, a standardization method was employed. A template with a fixed sentence structure was created, along with guidelines for completion. A trained individual extracted information from the original responses and filled in the template accordingly. A dual-review process was implemented to ensure consistency, and neutrality in language was maintained. This process ensured that the responses were structurally similar, providing a fair and unbiased basis for expert assessment.

Initially, a consistency analysis was performed on the ratings provided by three experts for 30 cases analyzed by ChatGPT. The Cronbach’s α coefficient was found to be 0.795, which suggests a relatively high degree of agreement among the expert ratings. As shown in **Table 4**, the mean scores (± SD) were 82.24 ± 9.933 for ChatGPT-4o, 79.83 ± 3.768 for GPs, and 86.35 ± 4.142 for APs. As case complexity increased, ChatGPT’s scores gradually decreased, with a mean score of 89.90 ± 2.936 for A-level cases, 81.22 ± 6.629 for B-level cases, and 76.12 ± 11.93 for C-level cases. The mean scores for GPs were comparable to those of ChatGPT-4o, but the score gradually decreased as the complex increased (A-level: 81.48 ± 3.084; B-level: 79.81 ± 4.236; C-level: 78.35 ± 3.622), but no statistically significant differences were observed among the groups. APs achieved the highest mean scores among the three groups, with 88.05 ± 3.538 for A-level cases, 84.77 ± 5.169 for B-level cases, and 86.09 ± 3.450 for C-level cases.

**Table 4:** Assessment scores and satisfaction levels of clinical responses by ChatGPT-4o, general practitioners, and attending physicians.

Subject of assessment	Mean score (Mean ± SD)	Satisfaction (100 - 85) (Number of questions)	Acceptability (84 - 60) (Number of questions)	Unacceptability (< 60) (Number of questions)
ChatGPT-4o	82.24 ± 9.933	17	11	2
	A:89.90 ± 2.936	9	1	0
	B:81.22 ± 6.629	4	5	0
	C:76.12 ± 11.93	4	5	2
GPs	79.83 ± 3.768	2	28	0
	A:81.48 ± 3.084	1	9	0
	B:79.81 ± 4.236	1	8	0
	C:78.35 ± 3.622	0	11	0
APs	86.35 ± 4.142	20	10	0
	A:88.05 ± 3.538	8	2	0
	B:84.77 ± 5.169	5	4	0
	C:86.09 ± 3.450	7	4	0

**Figure 3:**
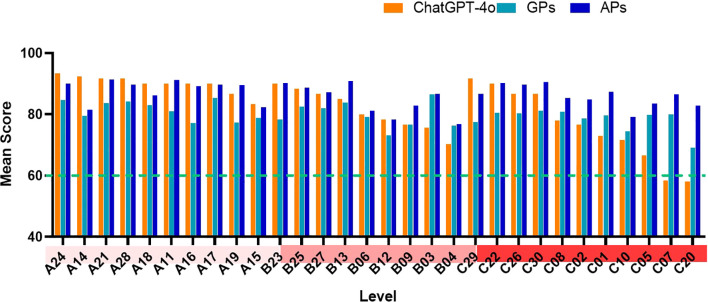
Mean scores for each case assessed by ChatGPT-4o, GPs, and APs. The horizontal axis represents the level of complexity [(A) simple, (B) moderate, and (C) complex] and question number, while the vertical axis depicts the mean score.

**Figure 4:**
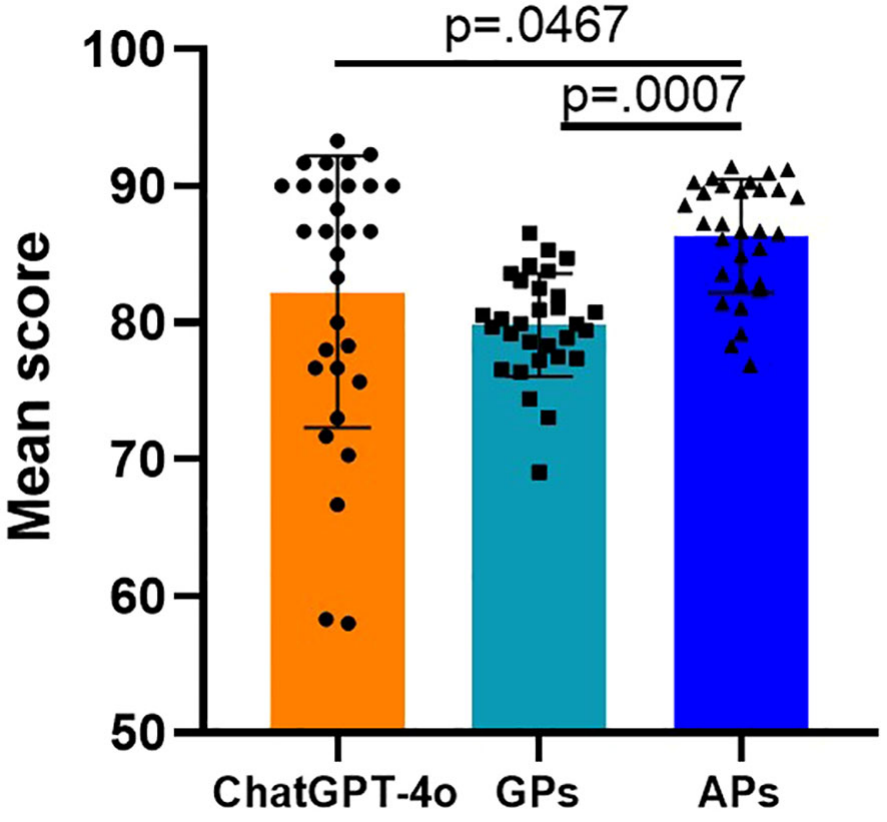
Mean scores for each group. The overall mean scores from the 30 clinical cases indicated that ChatGPT-4o achieved a mean score of 82.24 ± 9.933, which is comparable to that of GPs (79.83 ± 3.768), but lower than that of APs (86.35 ± 4.142). n = 30 for each group.

**Figure 5:**
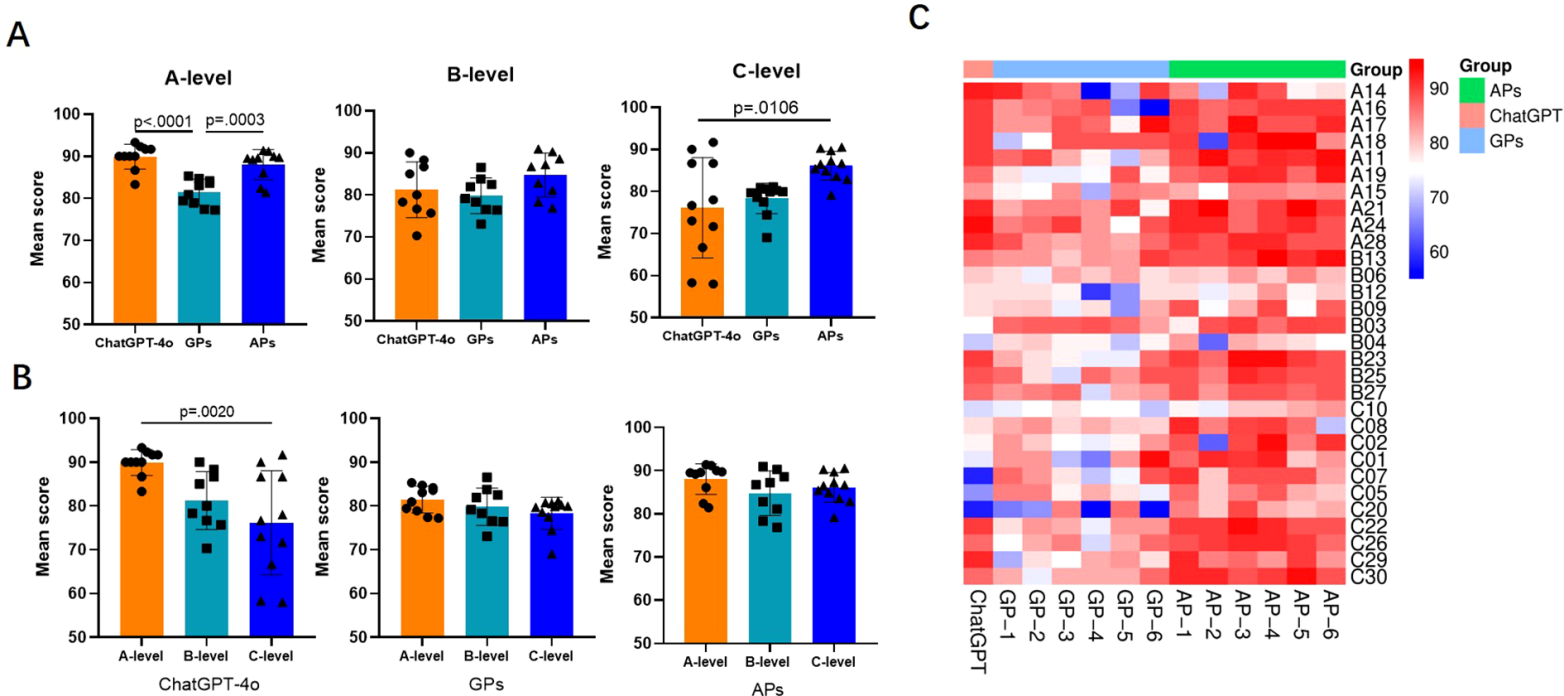
Mean scores of the groups for cases of different complexity. **(A)** Performance varied with case complexity. For A-level questions (n=10), ChatGPT-4o achieved the highest scores, while GPs had the lowest. There were statistically significant differences in scores between ChatGPT-4o and GPs, as well as between APs and GPs. For B-level questions (n=9), there were no significant statistical differences among the three groups. For C-level questions (n=11), ChatGPT-4o had the lowest scores, while APs achieved the highest. Significant statistical differences were observed between APs and ChatGPT-4o. **(B)** ChatGPT-4o demonstrated significant performance variation in A-level (n=10) and C-level (n=11), with mean scores declining markedly as case complexity increased. In contrast, GPs and APs showed no statistically significant differences in performance across the case categories. **(C)** This heatmap illustrates the scores of each individual across the three groups for each case question.

In terms of answer satisfaction (score ≥ 85), APs demonstrated the best performance, with 20 questions scoring above 85, indicating satisfaction with the answers, and another 10 questions scoring between 60 and 84, indicating acceptable answers. ChatGPT-4o provided 17 satisfactory answers, 11 acceptable answers, and 2 unacceptable answers. GPs provided only 2 satisfactory answers, while 28 answers were deemed acceptable. Among the three groups, only the ChatGPT-4o group contained unacceptable answers. **Figure 3** shown the mean scores for each case assessed by ChatGPT-4o, GPs, and APs. As shown in **Figure 4**, there was a significant difference in the overall mean scores of APs compared to both ChatGPT-4o and GPs. However, no significant statistical difference was observed between the mean score of ChatGPT-4o and GPs. **Figure 5A** illustrates that for A-level questions, ChatGPT-4o achieved the highest scores, while GPs had the lowest. There were statistically significant differences in scores between ChatGPT-4o and GPs, as well as between APs and GPs. For B-level questions, there were no significant statistical differences among the three groups. Interestingly, for C-level questions, ChatGPT had the lowest scores, while APs achieved the highest. Significant statistical differences were observed between APs and ChatGPT-4o. **Figure 5B** illustrates the performance differences among ChatGPT-4o, GPs, and APs across varying levels of case complexity. Notably, ChatGPT-4o demonstrated significant performance variation in categories A, B, and C, with mean scores declining markedly as case complexity increased. In contrast, GPs and APs showed no statistically significant differences in performance across the case categories. **Figure 5C** presents the scores for each individual question across the three groups, highlighting the performance differences in detail.

Within the C-level complex cases, ChatGPT’s performance exhibited considerable variability across different clinical scenarios. Four cases scored in the high range of 85–100 points, including fulminant type 1 diabetes, MODY3, mitochondrial diabetes, and diabetic ketoacidosis, suggesting that ChatGPT-4o demonstrates relative strength in managing special or atypical forms of diabetes. Five cases fell into the intermediate range of 60–85 points, encompassing diabetic kidney disease, diabetes with hyperlipidemia, diabetes with pancreatitis, diabetes with renal impairment, and diabetes with gastrointestinal dysfunction. In these cases, which involve coexisting complications or comorbidities, ChatGPT-4o’s recommendations were moderate, reflecting the additional complexity posed by multiple clinical factors. Two cases scored below 60 points, including a patient with sleep apnea and another with pancreatic cancer and impaired liver function (**Supplementary Table 2**). In these instances, ChatGPT-4o suggested glucose-lowering strategies that included drugs that are clinically contraindicated or should be used with caution. Although these medications are not strictly prohibited, their use requires careful consideration in the context of the patient’s overall clinical condition. These findings indicate that while ChatGPT-4o can handle typical and certain atypical diabetes cases well, its performance diminishes in highly complex scenarios involving severe comorbidities or organ dysfunction, highlighting areas where human clinical judgment remains essential.

## 4 Discussion

In our current study, we compared the abilities of ChatGPT-4o, GPs, and APs in formulating personalized glucose-lowering strategies for diabetic patients. We found that, overall, ChatGPT-4o performed comparably to GPs but was slightly worse than APs. In relatively simple cases classified as A-level, ChatGPT-4o demonstrated strong performance, significantly outperforming GPs. However, as case complexity increased, particularly in B-level and C-level, ChatGPT-4o’s performance gradually declined. Several recent studies have also highlighted limitations of AI and large language models in complex medical contexts. For example, analyses of LLM reasoning in open-ended clinical problem-solving have shown poor performance relative to physicians and tendencies toward inflexible pattern matching and overconfident outputs, particularly in scenarios requiring nuanced clinical judgment (18). However, an intriguing observation in our study is that the glucose-lowering regimens proposed by ChatGPT-4o for rare secondary diabetes cases, such as cases 22, 29, and 30, received high scores. We believe this finding can be explained by several factors. First, the diagnosis and management of these rare subtypes are largely guided by well-defined criteria and established guidelines, making them more knowledge- and rule-based tasks, which align well with the strengths of large language models in structured knowledge retrieval and pattern recognition. Second, ChatGPT has been trained on a broad corpus of medical literature, guidelines, and educational materials, allowing it to access and integrate information on uncommon conditions that general practitioners may encounter infrequently in routine practice. Third, the model provides highly consistent outputs that are not influenced by variability in individual clinical experience, whereas physicians’ performance in rare diseases may vary depending on prior exposure. In addition, the case vignettes used in this study typically presented characteristic clinical features, facilitating accurate knowledge matching by the model. Together, these factors may account for the comparatively higher scores observed for ChatGPT in rare diabetes subtypes. Importantly, this strength suggests potential applications of LLMs in clinical education and decision support, such as supporting trainee learning, reinforcing awareness of uncommon diagnoses, and providing structured reminders or preliminary recommendations. Nevertheless, we acknowledge that real-world management of these conditions often depends on contextual, psychosocial, and longitudinal factors that are not fully captured in vignette-based evaluations, and therefore such systems should be positioned as supportive tools rather than substitutes for clinical judgment.

Upon further analysis of the cases underperformed by ChatGPT-4o, we identified a key concern in ChatGPT-4o-proposed glucose-lowering strategies: the inability to effectively avoid certain contraindications. This limitation is particularly evident in medical scenarios requiring high precision and reliability, where inappropriate recommendations could lead to significant clinical consequences. For instance, in case 7, ChatGPT-4o recommended the use of metformin for a patient with severe obstructive sleep apnea and hypoxemia (SpO2 85%), which could significantly increase the risk of severe adverse effects such as lactic acidosis (19–22). Although cases of lactic acidosis due to metformin application are exceedingly rare, the mortality rate is around 30%-50% (22–26). Consequently, the U.S. Food and Drug Administration (FDA) recommends that these drugs be administered with caution in patients with hypoxemia. Another illustrative example is Case C20, in which a dipeptidyl peptidase-4 inhibitor (DPP-4i) was selected as the glucose-lowering therapy for an elderly patient with a prior history of pancreatic cancer, based on individual clinical characteristics. Although pancreatic cancer is not listed as a formal contraindication for DPP-4i, the drug label highlights an increased risk of pancreatitis. Both DPP-4i and glucagon-like peptide-1 (GLP-1) receptor agonists are incretin-based antidiabetic drugs. Incretin hormones, such as GLP-1, improve β-cell function and suppress glucagon secretion, thereby ameliorating hyperglycemia (27). However, incretin-based therapies are also known to exert pleiotropic effects on the exocrine pancreas, including stimulation of cellular proliferation and dysplastic changes (28). Previous observational studies have suggested that the use of DPP-4i in patients with type 2 diabetes may be associated with an increased risk of pancreatitis and pancreatic cancer (28–30). However, subsequent large cohort studies and meta-analyses have not consistently confirmed a causal relationship (31, 32). Nevertheless, from a clinical perspective, caution remains warranted when prescribing DPP-4i to patients with a history of pancreatic disease. In this case, selecting a DPP-4i as the first-line glucose-lowering therapy does not fully align with prudent, risk-aware clinical prescribing principles. Upon further analysis, we recognize that several factors may contribute to this limitation. First, the model’s training data, while extensive, may not include a sufficient number of real-world cases involving complex comorbidities or rare conditions, which could lead to gaps in recognizing contraindications. Second, its semantic reasoning capabilities, though advanced, may face challenges in synthesizing multiple pathological factors to provide a comprehensive risk assessment. Third, the coverage of the medical knowledge base, while broad, may not always be as detailed or up-to-date as professional clinical decision support systems, potentially leading to insufficient warnings for high-risk scenarios. However, recent research has demonstrated that the accuracy of LLMs in handling drug contraindications can be substantially improved through targeted training and knowledge integration. For example, by implementing a Retrieval Augmented Generation approach that combines LLMs with curated drug utilization data, models can better identify contraindications related to age, pregnancy, and concomitant medications. Such approaches highlight the potential for enhancing LLM-guided treatment recommendations and reducing the risk of prescribing errors in complex clinical scenarios (33).

Despite the less satisfactory performance on complex cases, the ChatGPT-4o exhibited certain capabilities during this trial. It performed consistently and excellently in simple cases (A-level). This indicates its potential to be a valuable supplementary tool for healthcare professionals, particularly in regions with limited resources, such as rural and low-income communities, where the scarcity of medical facilities and professionals can limit the effectiveness of diabetes management. Moreover, ChatGPT-4o can improve its accuracy through user prompts. For instance, in case 7, after we provided ChatGPT-4o with information about the FDA safety warning regarding metformin-induced lactic acidosis, it adapted its recommendations for the patient. While ChatGPT-4o still suggested metformin as a first-line treatment, it included alerts to monitor renal function (**Table 5**). This illustrates that ChatGPT-4o can adjust its recommendations based on specific clinical information provided by users.

**Table 5:** The initial and revised recommendations generated by ChatGPT-4o for a pharmacologic treatment plan.

34-year-old male patient with a diagnosis of diabetes mellitus for 1 month and previous respiratory sleep apnea hypoxemia. He was 180cm tall and weighed 187kg.BMI: 57.7 kg/m2. Liver function, renal function, blood lipids were normal, urine ketone body, urine protein was negative. SpO2 85%. Fasting blood glucose: 5 mmol/L. 2-hour postprandial blood glucose: 8.5 mmol/L, glycated hemoglobin: 6.3%. Fasting insulin level: 153.9 pmol/L (reference range 17.8-173 pmol/L). Please develop a reasonable glucose-lowering program for this patient.	
Previous	Update
Pharmacologic Therapy: Metformin: - Initial Dose: 500 mg once daily with food to minimize gastrointestinal side effects. Gradually increase to 500 mg twice daily, then to 1000 mg twice daily, as tolerated. GLP-1 Receptor Agonists (e.g., Liraglutide, Semaglutide): Effective for glucose control and weight loss. - Starting Dose: - Liraglutide: 0.6 mg daily for one week, then increase to 1.2 mg daily. If needed, further increase to 1.8 mg daily. - Semaglutide: 0.25 mg weekly for the first four weeks, then increase to 0.5 mg weekly. If needed, further increase to 1 mg weekly. - Benefits: Significant weight loss and improved glycemic control. SGLT2 Inhibitors (eg., Empagliflozin, Depagliflozin): Can be considered if additional glucose-lowering is needed and to aid in weight loss. - Starting Dose: - Empagliflozin: 10 mg once daily - Dapagliflozin: 5 mg once daily	Pharmacologic Therapy: Metformin: -Starting Dose: Begin with 500 mg once daily with food, gradually increasing to 1000 mg twice daily -Monitoring: Regularly monitor renal function, given the risk of lactic acidosis, especially in the context of obesity and potential renal impairment. GLP -1 Receptor Agonists Examples: Liraglutide, Semaglutide - Benefits Effective for weight loss and glycemic control. -Starting Dose: Initiate at a lower dose to minimize gastrointestinal side effects, and increase as tolerated. SGLT2 Inhibitors (Optional) Examples: Empagliflozin, Dapagliflozin. - Benefits: Weight loss, cardiovascular protection, and blood glucose control. -Monitoring: Watch for signs of urinary tract infections and dehydration. - Starting Dose: - Empagliflozin: 10 mg once daily. - Dapagliflozin: 5 mg once daily.

The development of personalized glucose-lowering strategies is crucial in diabetes management, taking into account factors such as age, comorbidities, kidney and liver function, and lifestyle. For newly diagnosed patients, general practitioners typically require significant time to formulate effective strategies. In our study, we observed that, after receiving the clinical data for each patient, general practitioners required approximately four minutes to formulate a strategy, whereas ChatGPT-4o completed the task in less than 30 seconds. This significant difference underscores a substantial improvement in clinical efficiency, particularly given the limited time available during healthcare visits.

Prior work has shown that ChatGPT perform well in guideline-based reasoning, standardized clinical examinations, and structured case simulations, often achieving scores comparable to medical trainees or non-specialist physicians (34, 35). However, most existing studies have primarily focused on relatively constrained or idealized tasks, such as insulin dose titration, forecasting glycemic trends, or providing personalized dietary recommendations (36–38). Direct comparisons between AI-generated treatment regimens and those developed by clinicians across varying levels of case complexity remain limited, particularly in real-world therapeutic decision-making.

Unlike prior studies emphasizing knowledge recall or isolated tasks, our study is the first to highlight ChatGPT-4o’s potential as a practical clinical support tool for healthcare providers, especially general practitioners, in the management of diabetes and the development of individualized glucose-lowering strategies. It demonstrated reliable performance in simple and moderately complex cases, offering significant advantages over traditional methods. Its higher scores in simpler cases underscore its utility for less experienced clinicians and medical students, providing 24/7 access to accurate information and enhancing clinical decision-making. It can effectively solve the problem of allocation and balance of medical resources. Furthermore, the latest version of ChatGPT-4o includes image and video processing capabilities, further enriching clinical consultations and addressing the challenges of resource allocation in healthcare. The advances of ChatGPT may prove to be a double-edged sword in the medical field. On the one hand, well-trained clinicians may find it helpful to have a digital assistant that can assist them to save time and improve efficacy. On the other hand, excessive reliance on large language model tools during clinical training may lead medical students to reduce essential hands-on practice and critical thinking exercises, which could adversely affect their professional development. Moreover, many diabetic patients lack sufficient medical knowledge and may become reliant on Internet and AI for improper diabetes management. However, the information provided by AI may be incorrect or misleading (9, 39–41), and patients may have difficulty evaluating the accuracy of the information they encounter. In the absence of a citation (42, 43), the applicant is unable to ascertain the reliability of the information provided by ChatGTP-4o. Nevertheless, the management of diabetes necessitates a comprehensive approach, wherein effective communication between physician and patient is of paramount importance. Physicians are responsible for providing detailed guidance to patients on the careful use of AI for medical purpose.

We acknowledge that the successful implementation of AI in clinical settings depends not only on technical performance but also requires careful consideration of trust, legal implications, and the physician’s role. While our study highlights the efficiency advantages of the proposed model, we agree that real-world applications must preserve physicians’ ultimate right of review. To ensure clinical utility, we envision positioning this model as a decision-support tool rather than a replacement for physician judgment. The AI’s outputs will be presented as recommendations, with clinicians retaining full authority to accept, modify, or override them. This approach aligns with existing frameworks for AI-assisted diagnostics, where human expertise remains central to patient care. Future iterations of ChatGPT should focus on enhancing its ability to manage complex clinical scenarios by improving algorithms to recognize contraindications and incorporating sophisticated risk assessment models raised by clinical experts. Continuous evaluation and training with real-world clinical data are essential for refining performance, updating the knowledge based on the latest medical findings, and incorporating feedback from healthcare providers. Additionally, integrating ChatGPT with electronic health records can enhance its utility in developing personalized treatment strategies. Efforts are still needed to establish regulations on AI usage to protect patient privacy and resolve ethical issues (44).

The present study is subject to several limitations. Firstly, the sample size is relatively limited, with only 30 typical cases selected, which may not fully capture the complexity and diversity in diabetes pathophysiology, warranting further studies with larger sample size and higher statistical power. The current sample size was determined based on practical constraints, including data availability and study feasibility. Importantly, despite the sample size, our results have demonstrated clear and meaningful trends that provide valuable preliminary insights into the capability of ChatGPT-4o in diabetes management. These findings lay a foundation for future studies with expanded cohorts to further validate and refine the observations. These preliminary findings provide valuable insights and lay the foundation for future large-scale validation studies. Secondly, although the evaluations were conducted by expert endocrinologists, there may still be a degree of subjectivity in the scoring, requiring further verification on the consistency and reliability of the evaluation criteria. Thirdly, despite the clinicians and experts being blinded to the study’s purpose and experimental groups, there may still be uncontrollable biases which could affect the results. Fourthly, it was conducted over a relatively short period of time, during which the long-term effects of the glucose-lowering strategies and the actual clinical outcomes for patients were not observed or evaluated. The identification and subsequent addressing of these limitations can provide a foundation for future research improvements. Fifthly, the study is the absence of patient feedback on the acceptability and satisfaction with the glucose-lowering strategies, which is a crucial indicator of treatment efficacy and adherence. Furthermore, the study design did not fully consider the influence of various factors on real-world clinical decisions, including environmental conditions, resource availability, and social support for patients. Our study specifically focused on pharmacologic treatment patterns to maintain a clear research focus that could be thoroughly examined within our dataset limitations. This targeted approach enabled deeper analysis of medication-related decisions while controlling for confounding variables. The exclusion of lifestyle modifications and complication management was based on several considerations: inconsistent documentation of these parameters in our electronic health records, the challenge of attribution since these elements are typically managed by multidisciplinary teams, and the lack of standardization in non-pharmacologic measures across institutions. We fully recognize that excluding these factors may lead to overestimation of the apparent effectiveness of pharmacologic interventions alone and may limit the generalizability of our findings to settings with different non-pharmacologic support systems. The identification and subsequent addressing of these limitations can provide a foundation for future research improvements.

## 5 Conclusion

The study suggests that in terms of diabetes management, ChatGPT-4o demonstrates stable performance in formulating glucose-lowering strategies for relatively simple cases. This implies ChatGPT-4o as a physician’s assistant tool, providing cues to develop rational glucose-lowering strategies for diabetic patients, and improving the efficiency of diagnosis and treatment. However, for more complex cases, ChatGPT-4o’s performance still requires further enhancement and optimization. Future research could focus on developing an AI model specifically optimized for medical applications, potentially by collaborating with healthcare professionals and training on databases specific to medical information. The powerful synergy of human expertise and judgment, enhanced by AI assistance, will result in a higher quality in diabetes management. The tight collaboration among patients, large language models, and healthcare professionals shall optimize diabetes management model.

## Data Availability

The original contributions presented in the study are included in the article/**Supplementary Material**. Further inquiries can be directed to the corresponding authors.
