# Supplementary material for: Efficacy of ChatGPT in personalized glucose-lowering strategy development: a clinician-based comparative study
Source: Front Endocrinol (Lausanne). 2026 Mar 11;17:1693381. doi: 10.3389/fendo.2026.1693381 (PMC13012919; doi:10.3389/fendo.2026.1693381)
Supplement: Supplementary file 2 [file Table2.docx]

**Table S2. ChatGPT-4o performance across C-level complex diabetes cases**

| **Score Range** | | **Number of Cases** | **Case Types** |
| --- | --- | --- | --- |
| 85–100 | 4 | | Fulminant type 1 diabetes MODY3 Mitochondrial diabetes Diabetic ketoacidosis |
| 60–85 | 5 | | Diabetic kidney disease Diabetes with hyperlipidemia Diabetes with pancreatitis Diabetes with renal impairment Diabetes with gastrointestinal dysfunction |
| <60 | 2 | | Diabetes with sleep apnea Diabetes with a history of pancreatic cancer and impaired liver function |
